# Influence of Geographical Location on Maternal-Infant Microbiota: Study in Two Populations From Asia and Europe

**DOI:** 10.3389/fcimb.2021.663513

**Published:** 2022-02-04

**Authors:** Yue Cheng, Marta Selma-Royo, Xin Cao, Marta Calatayud, Qi Qi, Jing Zhou, Lingxia Zeng, Izaskun Garcia-Mantrana, Maria Carmen Collado, Bei Han

**Affiliations:** ^1^School of Public Health, Health Science Center, Xi’an Jiaotong University, Xi’an, China; ^2^Department of Biotechnology, Institute of Agrochemistry and Food Technology-National Research Council (IATA-CSIC), Valencia, Spain; ^3^Department of Pediatrics, The Second Affiliated Hospital of Xi’an Jiaotong University, Xi’an, China

**Keywords:** maternal, neonate, gut microbiota, geographical location, *Bifidobacterium*

## Abstract

Early gut microbial colonization is driven by many factors, including mode of birth, breastfeeding, and other environmental conditions. Characters of maternal-neonatal microbiota were analyzed from two distinct populations in similar latitude but different continents (Oriental Asia and Europe). A total number of 120 healthy families from China (n=60) and Spain (n=60) were included. Maternal and neonatal microbiota profiles were obtained at birth by 16S rRNA gene profiling. Clinical records were collected. Geographical location influenced maternal-neonatal microbiota. Indeed, neonatal and maternal cores composed by nine genera each one were found independently of location. Geographical location was the most important variable that impact the overall structure of maternal and neoantal microbiota. For neonates, delivery mode effect on neonatal microbial community could modulate how the other perinatal factors, as geographical location or maternal BMI, impact the neoantal initial seeding. Furthermore, lower maternal pre-pregnancy BMI was associated with higher abundance of *Faecalibacterium* in maternal microbiota and members from *Lachnospiraceae* family in both mothers and infants. At genus-level, Chinese maternal-neonate dyads possessed higher number of phylogenetic shared microbiota than that of Spanish dyads. *Bifidobacterium* and *Escherichia/Shigella* were the genera most shared between dyads in the two groups highlighting their importance in neonatal colonization and mother-infant transmission. Our data showed that early gut microbiota establishment and development is affected by interaction of complex variables, where environment would be a critical factor.

## 1 Introduction

An adequate early microbial colonization is crucial for proper immunological and metabolic development (Clemente et al., 2012). Shifts during this process have been linked to an increased risk of non-communicable diseases (NCDs) such as allergies, metabolic disorders, as well as other long-lasting effects (Milani et al., 2017).

Neonatal gut colonization represents the *de novo* assembly of a complex microbial community, a process that is influenced by several environmental and also, host factors (Koleva et al., 2015). This complex process, still not well understood, and follows a time frame sequence depending on primary events at birth, such as delivery mode and feeding type, and subsequently undergoes a dynamic and non-random process during the development and maturation of infant gut microbiota (Sprockett et al., 2018). In this scenario, maternal microbiota represents one of the major determinants in the assembly of the offspring’s microbial profile (Dominguez-Bello et al., 2010; Ferretti et al., 2018). Maternal gut microbiota would reflect the impact of specific factors and other environmental exposures, such as diet, lifestyle and antibiotic exposure, which could be transferred to the neonate at birth and later, during lactation (Dominguez-Bello et al., 2010; Koleva et al., 2015). Thus, mode of delivery and type of feeding have been proposed as the main drivers and contributors that would shape the neonatal gut microbiota (Dominguez-Bello et al., 2010). Several studies have explored the impact of perinatal factors on early gut microbial colonization, but available information about the potential impact of geographical location, including diet, lifestyle and climate on maternal-neonatal microbiota composition, is still scarce. Previous studies have been shown a differential impact of specific factors on milk microbiota according to geographical location (Kumar et al., 2016), while the data on maternal-neonatal gut microbiota was not provided.

In this sense, geographical location has been highlighted as an important factor shaping the microbiota composition in the adult population due to differences in dietary patterns, cultural practices, and religion (Singh and Mittal, 2020). Thus, the current study aims to assess the impact of the maternal microbiota on the distribution of infant gut microbial communities at birth from two distinct populations.

## 2 Material and Methods

### 2.1 Study Design and Participants

The study comprised 194 mother-neonate pairs from two independent cohorts from China and Spain, corresponding to 87 dyads from each location.

#### 2.1.1 Chinese Cohort

Mother-infant pairs were recruited from a community-based randomized controlled trial conducted in Shaanxi Province, China (Latitude 34.34 and Longitude 108.940). All mother-infant pairs lived in Bin county, Shaanxi Province, China. Mothers were recruited after admission to the hospital for delivery. The eligibility criteria for the present study included: healthy pregnant women without complications such as hypertension, diabetes, or any other diagnosed disease, singleton pregnancies and healthy full-term neonates. All the participants were informed about the study and gave written consent. The study protocol was approved by the Ethic Committee of Xi’an Jiaotong University Health Science Center, China. Furthermore, the study was registered on the ClinicalTrials.gov platform, with the registration number NCT02537392.

#### 2.1.2 Spanish Cohort

Mother-infant pairs were randomly selected as a subset from a prospective and observational MAMI birth cohort recruited from 2015-2017 in the Spanish-Mediterranean area (Latitude 39.46 and Longitude 0.375), as detailed elsewhere (García-Mantrana et al., 2019). Clinical parameters of the mother and the newborn were obtained from medical staff’s clinical records at the hospital. The inclusion criteria included healthy pregnancies without diagnosed disease, and mothers older than 18 years of age. The exclusion criteria were the non-compliance with any of the inclusion criteria and, pro- and prebiotic treatment or the use of any other medication and drugs. Women were enrolled at the end of gestation and with follow-up participation during the first year of life. All participants received oral and written information about the study and written informed consent was obtained from all the families. The study protocol was approved by the Hospital Ethics Committees (Hospital Universitario y Politécnico La Fe and Hospital Clinico Universitario de Valencia). The study is registered on the ClinicalTrial.gov platform, with the registration number NCT03552939.

### 2.2 Biological Samples

In both cohorts, maternal fecal samples were collected by trained personnel prior birth. In the newborns, the first-pass fecal samples were collected at birth and within the first 24 hours. Both samples were stored in pre-numbered sterile tubes and immediately stored at -80°C until further analysis.

### 2.3 DNA Extraction and 16S rRNA Amplicon Sequencing

#### 2.3.1 Chinese Cohort

Total DNA was extracted from approximately 200 mg of fecal sample by using the QIAamp Fast DNA Stool Mini Kit (51504, QIAGEN, Germany), according to the specifications from the manufacturer. DNA concentration and purity were detected on NanoDrop2000 (ThermoFisher, USA) and 1.0% agarose gel electrophoresis.

#### 2.3.2 Spanish Cohort

Total DNA was extracted from the fecal material (approx. 50-100 mg) using the Master-Pure DNA extraction Kit (Epicentre, Madison, WI, US) following the manufacturer’s instructions with the following modifications: samples were treated with lysozyme (20 mg/mL) and mutanolysin (5U/mL) for 60 min at 37°C and a preliminary step of cell disruption with 3-μm diameter glass beads for 1 min at 6 m/s by a bead beater FastPrep 24-5G Homogenizer (MP Biomedicals) as described elsewhere (García-Mantrana et al., 2019). Purification of the DNA was performed using DNA Purification Kit (Macherey-Nagel, Duren, Germany) according to manufacturer’s instructions. DNA concentration was measured using Qubit^®^ 2.0 Fluorometer (Life Technology, Carlsbad, CA, US) for further analysis.

#### 2.3.3 Sequencing

The V3-V4 region of the 16S rRNA sequence was amplified using a specific primer (Klindworth et al., 2013; Michelsen et al., 2014). DNA libraries were performed with the amplification of the V3-V4 variable region of the 16S rRNA gene. A multiplexing step was conducted by the NextEra XT Index Kit (FC-131-2001) (Illumina, San Diego, CA, United States). Amplicons were checked with a Bioanalyzer DNA 1000 system (Agilent Technologies, Santa Clara, CA, United States) and libraries were sequenced using a 2x300bp paired-end run (MiSeq Reagent kit v3) on an Illumina MiSeq platform according to manufacturer instructions. Controls during DNA extraction and PCR amplification were also included and sequenced. The sequenced data were submitted to SRA with the accession number of PRJNA637167 and PRJNA614975.

### 2.4 Bioinformatic and Statistical Analyses

Raw sequences from both locations were processed in the same manner but independently. The resulting taxonomical tables were then merged at genus level to avoid the potential bias at amplicon sequence variant level (ASV). The paired-ends reads were merged using FLASH v1.2.7 (Magoč and Salzberg, 2011). Then, Deblur method of Quantitative Insights into Microbial Ecology2 (QIIME2) software (v2020.1) (Bolyen et al., 2019) was used to extract taxonomical composition from the sequencing reads with the standard recommended options for the filtering and denoising process as well as in the chimeral identification and removal. Taxonomic assignment was conducted using the Silva v138 database (Yarza et al., 2008) and the available pretrained naive Bayes classifier (Bokulich et al., 2018; Kaehler et al., 2019). Final QIIME2 objects were imported to Rstudio environment for further quality filtering and statistical analysis. To manage the potential contaminants, all sequences from the negative controls from both, Spain and China data sets, were obtained and included in the pipeline. Furthermore, the *decontam* package (Davis et al., 2017) in R environment (R Core Team, 2019) was used to determine the presence of potential contaminants-related sequence and they were removed from the final table. Additionally, samples with less than 1000 sequences were removed from the final data set (n=0).

Alpha diversity indices, including those for determination of richness (Chao1), diversity (Shannon) was obtained through the phyloseq package (McMurdie and Holmes, 2013) after the rarefaction of the tables to the minimum reads per sample (3947 reads). Differences in alpha-diversity index were performed using Mann-Whitney test considering a *p<0.05* as significant. Beta diversity analysis were performed based on Bray-Curtis distance using also phyloseq package. Adonis test from vegan package (Oksanen et al., 2019) was used to assess the association between gut microbial community composition and studied variables (Bäckhed et al., 2015). The analysis included the following variables for the study of maternal microbiota: geographical location, maternal age, maternal body mass index (BMI) and delivery mode. Principal Coordinate Analysis (PCoA) and Discriminant analysis of Principal components (DAPC) were performed to the visualization of the β-diversity similarities according to geographical location through the adegenet (Jombart and Ahmed, 2011), ggplot2 (Wickham, 2016) and vegan package.

Maternal and neoantal core genera were obtained through the microbiome package. A threshold of 0.01% in 95% of the samples and 85% was considered for the identification of maternal and neonatal microbial core, respectively. The core taxa were plotted using ggplot2 with a after log transformation of the data to facilitate the visualization. For the core genera the co-occurrence OTUs were verified. Spearman’s rank correlation was calculated and employed to identify the co-occurrence patterns among the relative abundance of core genera. R package “corrplot” (Wei and Simko, 2021) was used to plot the correlations matrix. For the analysis of the compositional differences between the studied variables two approaches were used. For the initial exploration, differences in the phyla and core genera were assessed by Mann-Whitney analysis on the centered log ration (CLR) normalization through the microbiome package (Lahti and Shetty). To control for multiple testing, false discovery rate (FDR) values were estimated by the Benjamini-Yekutieli method (Benjamini and Yekutieli, 2001) (referred as q-value). Besides this, Maaslin2 package (Mallick et al., 2021) was used to performed multivariate analysis for the effect of geographical location in the relative abundance of microbial genera in both maternal and infant microbiota including the potential influential covariables (maternal BMI, delivery mode and age). For multivariate analysis in Maaslin2 those genera that appear less than 3 times in at least 10% of the samples were removed (remaining 181 genera). Mann-Whitney test was also used for testing the differences between the bacterial counts, for total bacteria and *Bifidobacterium* genus, in infant samples measured by quantitative PCR using GraphPad prism software (GraphPad Prism version 8.4.3 for Windows, GraphPad Software, San Diego, California USA, www.graphpad.com).

### 2.5 Bacterial Quantification by Quantitative PCR Analysis

A small subset of infant samples (n=67) according to DNA availability (China n=36; Spain n=31) were used for the specific bacterial count determination by the qPCR. Total bacterial and *Bifidobacterium* genus counts were measured by quantitative system based on the amplification of specific 16S rRNA gene region by use of Light Cycler 480 Real-Time PCR System (Roche, Basilea, Switzerland). Primers used were for total bacteria (Fwd: 5’-CGTGCCAGCAGCCGCGG-3’, Rv: 5’-TGGACTACCAGGGTATCTAATCCTG-3’) and for *Bifidobacterium* genus (5’-GATTCTGGCTCAGGATGAACGC-3’; Rv: 5’-CTGATAGGACGCGACCCCAT-3’) (Gueimonde et al., 2004; Farhana et al., 2018). Reaction mixture consisted in SYBR Green I master mix (Roche, Basilea, Switzerland), 0.25 µM of each specific primer set and 1 µl of DNA. Melting curves were also assessed to test the specificity of the reaction. Standard curves for the specific targeted bacterial group were generated using Ct values and the calculated gene copy numbers were determined based on the fragment amplification length.

## 3 Results

### 3.1 Study Population

A total of 194 pregnant women from China and Spain were included in the study, of which 74 dropped out. Reasons for the drop-out of participants included the following: no longer fulfilling the inclusion and exclusion criteria (n=31), withdrawal of informed consent (n=25), missing data of subjects (n=11) and other reasons (n=7). Finally, 120 mother-neonate dyads (240 fecal samples) were suitable for analysis. The characteristics of the subjects participating in the study are listed in **Table 1**. Chinese mothers were younger than the Spanish ones (a median of 27 vs 31.6 years) (*p=0.001*). Similarly, a slight difference was observed in gestational age showing lower gestational age in Chinese population than in Spain, although both were term deliveries (38.8 vs 39.6 weeks) (*p<0.001*). No differences were observed in the other recoded clinical characteristics.

**Table 1:** The demographic and birth characteristics of subjects from China and Spain.

	China (n=60)	Spain (n=60)	*P*
Maternal characteristics			
Maternal age (y)	27.0 (21.3-37.3)	31.6 (22.3-40.1)	0.001*
BMI	21.3 (16.6-28.3)	22.5 (16.4-29.7)	0.110
Infant characteristics			
Gestational age	38.8 ± 1.1	39.6 ± 1.1	>0.001*
Male	39/65%	35/58.3%	0.453
Female	21/35%	25/41.7%	
Vaginal delivery	45/75%	50/83.3%	0.261
Non-vaginal delivery	15/25%	10/16.7%	
Birth weight (g)	3205.7 ± 399.5	3347.5 ± 440.3	0.067

*P < 0.05.

### 3.2 Sequencing Summary and Gut Microbiota Characteristic in Maternal-Neonatal Dyads

**Figure 1:**
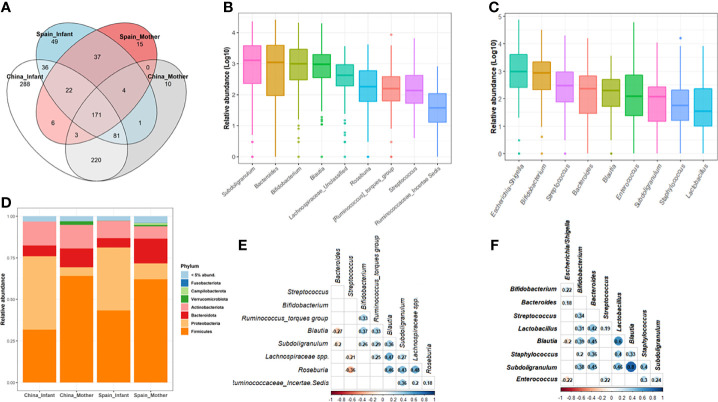
Microbial composition of maternal-neonatal microbiota in both populations, China and Spain. **(A)** Venn diagram of the shared genera between mothers and neonates as well as both populations. **(B, C)** The relative abundance distribution of the core genera in the whole maternal **(B)** and neonatal group **(C)**. Microbiota data at genus level was transformed to log10-values for plotting [log (x+1)]. **(D)** The microbial composition of the whole population at phylum level according to both geographical location and mother/infant category. Phyla with a relative abundance lower than 5% were groups as “Others”. **(E, F)** Co-occurrence patterns among the core genera across the 120 maternal **(E)** and neonatal **(F)** samples determined as Spearman’s correlation. Data were transformed to relative abundance for correlations analysis. Only the significant relations are colored.

After data filtration and chimera removal, the 120 mother-neonate dyads dataset contained 7,218,087 reads (min-max reads: 3947- 93206). The resulted phyloseq object consist in a total of 944 different genera catalogued in the Silver138 database (**Figure 1**). Furthermore, the retrieved genera were distributed among 37 phyla and 364 families. Among genera, 171 were shared between Chinese and Spanish population (18.11%) (**Figure 1A**). The microbial core at genus level of maternal and neonatal microbiota were composed by 9 genera in both populations (**Figures 1B, C** and **Supplementary Data**, **Table S1**).

For the brief characterization of the maternal-neonatal gut microbiota from China and Spain, the most abundant bacterial phyla are presented in **Figure 1**. In 120 pregnant women, Firmicutes (average 63.47%) and Bacteroidetes (average 13.45%) were the most dominant phyla, and *Bacteroides* (8.33%), *Subdoligranulum* (8.50%) and *Bifidobacterium* (8.62%) were the most abundant genera on average (**Supplementary Data**, **Table S1**). Regarding neonatal microbiota, Proteobacteria (41.24%) and Firmicutes (37.73%) were the dominant phyla, and the main genera were *Escherichia/Shigella* (18.35%) and *Bifidobacterium* (9.41%) (**Figure 1D**).

### 3.3 Impact of Location on Maternal Gut Microbiota

There were nine core genera present in 120 mothers which is composed by *Subdoligranulum, Bifidobacterium, Bacteroides, Blautia, Lachnospiraceae spp., Roseburia, Streptococcus, Ruminococcus torques group and Ruminococcaeae* unclassified spp. (**Figure 1B**; **Supplementary Data**, **Table S1**). The collective core was overwhelmingly dominant (abundance >30%) in almost two thirds of the subjects (64%) but showed dramatic variations in the relative abundance of each genus in different cohorts, regardless of geography, lifestyle, and ethnicity (**Supplementary Data**, **Figure S1**). Spearman’s rank correlation test was performed to identify the co-occurrence patterns among the 9 core genera (**Figure 1E**). In general, correlations were found among the core genera components from related genera such as *Lachnospiraceae* and *Ruminococacceae* groups and *Subdoligranulum*, *Blautia* and *Roseburia* with a rho ranged 0.2-0.48. A negative association was found between some of these genera such as *Blautia* (rho=-0.27, p=0.003) and *Suboligranulum* (rho=-0.20, p=0.031) with *Bacteroides* genus.

**Figure 2:**
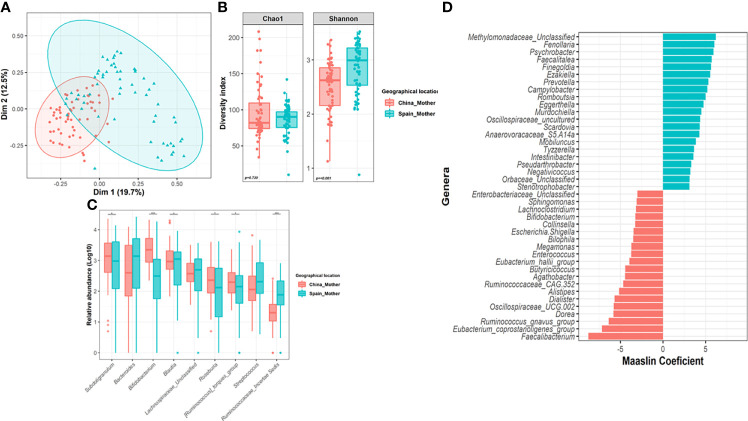
Impact of geographical location on the maternal gut microbiota. **(A)** Principal Co-ordinates Analysis (PCoA) analysis based on Bray-Curtis distance at genus level according to location. **(B)** Differences in alpha-diversity of the maternal microbiota (Shannon and Chao1 index) according to location. Significance of the differences were assessed by Mann-Whitney test. **(C)** Comparison of core genera relative abundance between mothers from both locations. Significance was assessed by Mann-Whitney test after centered log ration transformation (CLR). Microbiota data were transformed to log10-values for plotting. **(D)** Results from the multivariate Maaslin2 analysis showing the differences in terms of microbial composition according to location adjusted by maternal age and body mass index. Only those genera that appeared more than 3 times in at least 10% of samples were included in the analysis. To facilitate the visualization, only those genera with a coefficient higher than 3 was plotted (complete list in **Table S2**). (*P < 0.05; ***P < 0.001).

Despite the common core, significant differences were found in maternal microbiota between China and Spain (Adonis, F.model = 19.30; R2=0.138, *p=0.001*) which were visualized by PCoA (**Figure 2A**). In terms of alpha-diversity, spanish mothers (M-ES) showed higher richness based on Shannon (*p<0.001*) index than chinesse mothers (M-CN) group, but no differences were found in microbial diversity (*p=0.730*) (**Figure 2B**).

Compositional analysis revealed that China mothers harbored a microbiota enriched in Actinobacteriota (*p=0.003*, *q=0.008*) and Verrucomicrobiota (*p=0.006*, *q=0.019*) phyla while mothers from Spain showed higher relative abundance of Proteobacteria (*p=<0.001*, *q<0.001*) and Bacteroidota (*p=0.008*, *q=0.019*). Among the nine core genera present in 120 mothers, the maternal microbiota core had significant difference between the two countries, for *Bifidobacterium (p<0.001, q<0.001), Blautia (p=0.006, q=0.012), Subdoligranulum (p=0.014, q=0.021)*, *Roseburia (p=0.006, q=0.012)* and those groups from *Ruminococcaceae* family (**Figure 2C**). Indeed, the adjusted multivariate analysis revealed that Chinese mother harbored higher relative abundance of *Faecalibacterium* (*q<0.001*), *Ruminococcus gnavus* group (*q<0.001*), *Eubacterium_coprostanoligenes_*group (*q<0.001*) or *Enterococcus* (*q<0.001*), among others compared to Spanish mothers (**Figure 2D**). However, *Finegoldia* (*q<0.001*), *Ezakiella (q<0.001)*, *Prevotella (q<0.001)* or *Campylobacter* genera had significantly lower relative abundance in the M-CN group than in the M-ES group.

Furthermore, the multivariate analysis revealed that maternal BMI was significantly negative associated to *Eubacterium eligens* group (Coef=-0.84, *q=0.020*), *Lachnospira* (Coef=-0.78, *q=0.021*) and *Oscillonospiraceae* UCG005 (Coef=-0.93, *q=0.023*) after adjustment by covariates (**Supplementary Data**, **Table S2**).

### 3.4 Impact of Location and Delivery Mode on Neonatal Gut Microbiota

There were nine core/predominant genera present in 120 neonatal microbiota including *Escherichia/Shigella*, *Bifidobacterium*, *Streptococcus*, *Bacteroides* and *Blautia* as the most abundant/prevalent (**Figure 1C** and **Supplementary Data**, **Table S1**). Indeed, these nine collective cores were dominant (abundance >30%) in more than 73% of the subjects (**Supplementary Data**, **Figure S2**). Results of Spearman’s rank correlation test showed that except for the *Eschericha/Shigella* genus, most of the other core genera were positively correlated with each other (**Figure 1F**).

**Figure 3:**
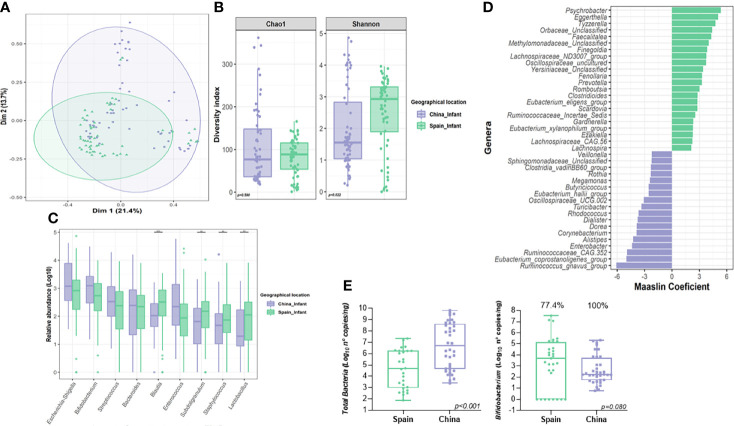
Influence of geographical location on the neonatal gut microbiota. **(A)** Principal Co-ordinates Analysis (PCoA) analysis based on Bray-Curtis distance at genus level according to location. **(B)** Differences in alpha-diversity of the neonatal gut microbiota (Shannon and Chao1 index) according to location. Significance of the differences were assessed by Mann-Whitney test. **(C)** Comparison of core genera relative abundance between neonates from both locations. Significance was assessed by Mann-Whitney test after centered log ration transformation. Microbiota data was transformed to log10-values for plotting. **(D)** Results from the multivariate Maaslin2 analysis showing the differences in terms of microbial composition according to location adjusted by maternal, body mass index and delivery mode. Only those genera that appeared more than 3 times in at least 10% of samples were included in the analysis. To facilitate the visualization, only those genera with a coefficient higher than 2 was plotted (complete list in **Table S3**). **(E)** Comparison of the quantitative analysis of total bacterial and *Bifidobacterium* counts expressed as log_10_ (number of copies of 16S rRNA gene for each group/ng of DNA). Significance of the differences were assessed by Mann-Whitney test on the log-transformed data. (**P < 0.01; ***P < 0.001).

Differences in neonatal microbiota between countries were revealed by PCoA (**Figure 3A**) (Adonis, F.model=4.36, R^2^=0.034, *p=0.001*). In terms of alpha-diversity, higher microbial richness was observed in Spanish infants compared to those born in China (*p=0.022*) while no differences were found in diversity measured as Chao index (*p=0.590*) **(****Figure 3B**). Among nine core genera present in 120 neonates, Mann-Whitney test on the CLR data, revealed that *Subdoligranulum* (*p<0.001*, *q=0.001*), *Staphylococcus* (*p=0.001*, *q=0.001*) and *Lactobacillus* (*p<0.001*, *q=0.001*), showed a significant difference in terms of relative abundance between the two countries (**Figures 3C, D**). However, in the multivariate analysis, the relative abundance of *Ruminococcus gnavus* group (*q=0.001*), *Enterobacter (q<0.001)* and *Corynebacterium* (*q<0.001*) among others, were observed higher in Chinese neonates compared to Spanish ones, while *Eggerthella* (*q<0.001*), *Finegoldia* (*q<0.001*) or *Prevotella* (*q=0.007*) were all significantly lower in Chinese neonates compared to Spanish ones **(****Figures 3C, D**; **Supplementary Data**, **Table S3**). Furthermore, Chinese neonates group showed higher total bacterial counts (*p=0.001*) than those born from Spain group (**Figure 3E**). Indeed, while no differences in terms of total counts of *Bifidobacterium* were observed according to country (*p=0.542*); higher number of infants from Spanish group showed a *Bifidobacterium* counts lower than the detection values (100% positive samples in China group compared to 77.4% of Spanish samples) (**Figure 3E**).

Delivery mode also showed an effect in the overall β-diversity of the neonatal microbiota at delivery (F.model=3.36, R^2^=0.026, *p=0.002*). The multivariate analysis adjusted by nationality demonstrated that vaginally delivered neonates were associated with an increased relative abundance of *Escherichia/shigella* genus (*q=0.001*) (**Supplementary Figure S2** and **Table S2**). However, C-section neonates showed a higher abundance of *Veillonella* (*q=0.001*) genus (**Supplementary Data** and **Figure S3** and **Table S3**). Due to the potential impact of geographical location on specific genera, the microbiota similarity among Chinese neonates (N-CN) and Spanish neonates (N-ES) groups in vaginal and cesarean delivery was assessed separately (**Supplementary Data**, **Figure S3**). Among 120 neonates, within the same delivery mode, the gut microbiota composition showed a significant difference between the two countries (N-CN *vs.* N-ES, Adonis, vaginal delivery, F=2.71, R^2^=0.028, *p=0.006*; cesarean delivery, F=3.87, R^2^=0.14, *p=0.001)*. Within the same country, while there was no difference observed according to delivery mode in children born in Spain (vaginal *vs.* cesarean, Adonis, N-ES; F.model=1.71, R^2^=0.029, *p=0.062*), delivery mode showed an impact in the overall microbial b-diversity in samples from children born in China (vaginal *vs.* cesarean, Adonis, N-CN; F=3.83, R^2^=0.062, *p=0.001)*.

The multivariate analysis adjusted by the previously stated covariables also revealed that some genera from neonatal microbiota were negatively associated with maternal BMI, including *Lactobacillus* (*q=0.035*), *Lachnospiraceae*_NK4A136_group (*q=0.003*) and *Ruminococcus* (*q=0.023*), among others (**Supplementary Data**, **Table S3**).

### 3.5 A Genus-Level Phylogenetic Shared Microbiota in Maternal-Neonate Dyads

**Figure 4:**
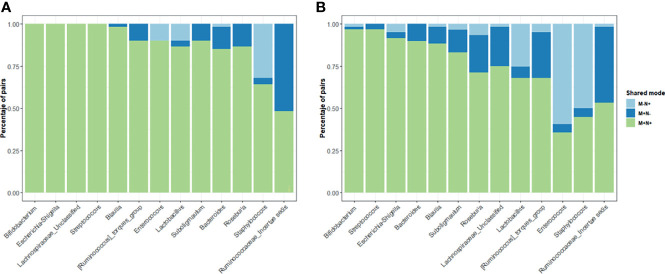
Distribution of the shared genera between mother-neonate dyads Only those genera from both maternal and neonatal core were included in the study of shared genera in China **(A)** and Spain **(B)** populations. Three modes of genera presences/absence were established: M+N+ (genera present in both mother and infant of each dyad), M+N- and M-N+ (genera present in only mother or only neonate, respectively). The percentage of dyads in each share mode were calculated for each core genera.

For the shared genera between maternal-neonate dyads, simultaneously detected genus at the same maternal-neonate dyads was regarded as a positive shared genus (**Figure 4**). Three shared modes were established, M+N+ (genus exists both in the mother and her neonate); M+N- (genus exists only in the mother, not in her neonate); M-N+ (genus does not exist in the mother, only in her neonate).

When the maternal and infant core genera was analyzed, *Bifidobacterium*, *Escherichia/Shigella* or *Streptococcus* were observed in the M+N+ mode in more than 95% of the dyads indicating stable and important functions of those two genera in the gut microbiota for the maternal-neonatal microbial relation. Some genera from the maternal-neonatal core were observed only in mothers in both populations and were rarely observed in an infant without being in the mother including *Blautia*, *Subdoligranulum* or *Lachnospiraceae* spp. and unclassified genera from *Ruminococcaceae* family suggesting a potential maternal transference to the offspring microbiota during the delivery. On the contrary, *Staphylococcus*, specially in Spanish population, showed higher presence of the M-N+ pattern.

## 4 Discussion

Human gut microbiota co-evolved with the host and participates in maintaining local and distant physiological homeostasis. During the birth process and immediately after, newborns experience vast contact with maternal and surrounding environmental microorganisms, which serve as early inoculation sources affecting short and long-term health outcomes (Bäckhed et al., 2015). Certain features of the infant gut microbiota, such as reduced diversity or atypical composition, have been linked to diseased states in the following childhood or adulthood, including asthma, inflammatory bowel disease or metabolic disorders (Sevelsted et al., 2015; Milani et al., 2017).

Bacterial transference from the mother at birth is one of the main contributors to neonates’ first contact with bacterial communities. Multiple factors can affect the “seeding” process and the maternal transference of microorganisms to the newborn, such as mode of delivery, gestational age at birth, antibiotic exposure, maternal diet, neonate feeding mode, environment (family lifestyle and geographical location) or host genetics (Milani et al., 2017). The birth mode is one of the most studied parameters affecting neonatal microbiota acquisition, but population or region-specific factors influencing the microbiota composition of newborns are largely unexplored. Our study aims to assess the impact of the maternal microbiota on the distribution of infant gut microbial communities at birth from two distinct populations, and we proposed a hypothesis that differential microbiota patterns associated with distant geographical locations are also transferable to the newborns.

Different geographical locations are related to specific dietary, behavioral, climatic and economic factors that shape the human microbiota (Benezra et al., 2012). Previous research has shown significant microbiota composition variations in healthy individuals from different races and ethnicities belonging to different or proximal geographical areas (Gupta et al., 2017). The microbiota of children (age from 1-6) of Europe and rural Africa (De Filippo et al., 2010), children (age from 9-14) of urban Bangladesh and suburban United States (Lin et al., 2013), infants (1 year of age) of Caucasian and South Asian descent (Stearns et al., 2017) significantly differ. We observed an effect of geographical location in microbiota profiles of first-pass neonatal and maternal fecal samples from China and Spain.

In our study, nine core genera present in 120 mothers constituting a genus-level phylogenetic core where location (country) is the main contributor of the microbiota variations. Among the core genera, Chinese women had a higher abundance of *Bifidobacterium* and *Subdoligranulum* than their Spanish counterparts, likely promoting maternal-infant transmission at birth and a primary ethnographic impact. The role of birth mode on bacterial transmission has been widely reported, however, recent studies described stable meconium microbiota structure regardless of mode of delivery (Chu et al., 2017). Liu reported three distinct types of meconium samples not influenced by the delivery mode (Liu et al., 2019). In our results, regarding the diversity of neonates’ gut microbiota, it seems geographical location has a higher influence than delivery mode. Thus, the first neonatal microbiota acquisition is a multifactorial process that needs to consider multiple factors such as birth mode, geographical location, maternal diet, environmental parameters, drugs/antibiotic exposure, parental contact, and many others still unknown. These results point out the need for further research on the maternal transmission of microbiota to the newborn.

Different studies have determined the microbiota composition of the first pass feces in neonates, showing that *Bifidobacterium*, *Enterobacteriaceae*, *Enterococcaceae*, and *Bacteroides*-*Prevotella* were prevalent genera (Hansen et al., 2015). Our microbiota in first-pass fecal sample results from 120 neonates are in line with published data, with a core microbiota composed mainly by *Escherichia/Shigella*, *Bifidobacterium*, *Enterococcus*, *Streptococcus* and *Bacteroides* in both geographical locations, however with specific differences in relative abundances between the Spanish and Chinese groups. Differences in first-pass neonatal fecal microbiota would be related to the sampling time as samples from Spain were collected immediately after birth in the delivery room while samples from China were collected during first 24h where the neonate would be exposed to other bacteria. In both cases, the microbial alpha-diversity indexes were comparable to those from the maternal gut and potentially higher than infant gut microbiota during first days, as reported previously (Mueller et al., 2017; Selma-Royo et al., 2020).

In our study, *Bifidobacterium* is the only bacteria shared in almost 100% of the maternal-neonate dyads at birth with no ethno-geographical difference. *Bifidobacterium* is established in the neonatal gut within the first days after delivery, but antibiotic exposure or reduced maternal transference can lag in engrafting on neonatal gut communities. In addition, it has been reported that delayed or disturbed colonization of *Bifidobacterium* during infancy increases the risk of suffering childhood diseases such as asthma or allergies but could also modulate the host health in adulthood by affecting the programming and development of the immune system and the protective functions of epithelial cells (Bailey et al., 2014). Previously, *B. breve*, *B. infantis* and *B. longum* were the most found species in an infant’s gut (Grönlund et al., 2011; Mikami et al., 2012; Matsuki et al., 2016). Toda et al. reported 11 isolates of *B. pseudocatenulatum* from a total of 48 isolates of feces and oral fluids of Japanese vaginally born neonates (Toda et al., 2019); in particular, the authors reported a *B. pseudocatenulatum* with high DNA homology in fecal samples of mothers, and oral cavity and fecal samples of the newborns, indicating possible fecal/oral route of transmission during birth. Thus, in vaginally delivered infants, acquisition of *Bifidobacterium* seems to occur *via* an oral route, which would explain the lower prevalence of *Bifidobacterium* species in neonates from cesarean section births.

Previously, Mikami et al., found a significant association between gut maternal colonization by *B. bifidum* and *B. breve* and increased numbers of *Bifidobacterium* species in the infant gut at 1 and 6 months (Mikami et al., 2012). Likely, observed changes in our study could be maintained during a critical window of an infant’s immune development (Milani et al., 2017). Recently, Yang et al., studied the fecal microbiota and bifidobacterial communities of 111 healthy Chinese volunteers of varying age profiles, from childhood (1-5 years) to long-lived individuals (≥90 years) (Yang et al., 2020). *Bifidobacterium* species have been isolated from human milk samples, therefore maternal transmission through breastfeeding may be a primary source for the infant gut (Turroni et al., 2019). In our study, the presence of *Bifidobacterium* in first-pass feces may derive from another source than breastmilk. Remarkably, Chinese neonates showed a higher number of positive samples for *Bifidobacterium* genus than Spanish samples. The first-pass fecal samples were obtained during the first 24 hours of life in Chinese population; therefore, some influence of early breastfeeding could be present in the sample. Similarly, Chinese infants showed higher total bacterial counts than the infants form Spain, which could also indicate a potential impact of the first 24h.

On the other hand, the genus only found in neonatal samples are likely acquired by different sources than the mother (M-N+), such as *Staphylococcus* or *Enterococcus* in Spanish neonates. The increase of *Enterococcus* in children has been related with food sensitization (Chen et al., 2016), however, the consequences of differential gut microbiota acquisition and temporal patterns of microbiota evolution in our cohorts would require longitudinal studies assessing specific health outcomes. Compared to the Chinese cohort, the Spanish cohort has more M-N+ samples, likely due to different birth practices (e.g., wiping or aspiring the neonates’ mouth, time from birth until breastfeeding or disinfecting products.) or differential environmental microbiota. Adams et al., showed that geography and building type shape indoor environmental microbes (Adams et al., 2015). Lax et al., showed that bacterial communities on patients’ skin and hospital room surfaces became similar throughout a patient’s stay (Lax et al., 2017), which involves a two-way sharing process between humans and the indoor environment. Indoor hospital microbiota would likely be different between China and Spain, influencing the microorganisms found in both populations. The presence of more shared genera (M+N+) between Chinese mothers and newborns indicates a more efficient vertical transmission of the microbiota, which can be associated to external/environmental influences (e.g., diet, birth practices) or inherent factors from both host (e.g., genetics) or microbiota (e.g., structure, resilience) and deserves further attention.

The acquisition and early maturation of infant microbiota is not well understood despite its likely influence on later health. In our study, the microbiota overlap between the maternal fecal sample and neonatal first-pass feces was minimal, while the similarity between paired maternal-neonate gut microbiota was more pronounced. The sources of a large proportion of infant microbiota could not be identified in maternal microbiota, the sources and function of seeding of infant gut microbiota remain to be elucidated.

This study has important strengths and also, some limitations. We had a bigger sample size compared to the prior studies of maternal-neonatal gut microbiota at birth time, and it includes gut microbiota data directly from maternal-neonatal dyads in two geographical locations, where the ethnicity, geographical location, maternal diet, and environmental parameters were all in consideration. Other limitation would be associated with the low-biomass present in the samples from first pass neonatal gut microbiota and the potential contaminant removal that would introduce some bias in the data and interpretation. It is difficult to completely control for certain effects, particularly different birth practices for the first-pass fecal samples. Despite those limitations, our main aim was to show the impact of the environment on the maternal-neonatal microbiota. In our study, geographical location would imply different diet, lifestyle, and genetic background, among other parameters. Therefore, it is so difficult to distinguish the main contributors to the maternal-neonatal variation and specific information on diet and lifestyle was not fully completed and not included in the study. Thus, further studies targeted to identify the main contributors are needed.

In all those potential influences, we verified that differential microbiota patterns associated with distant geographical locations are also transferable to newborns. *Bifidobacterium* is the only bacteria shared in almost 100% maternal-neonate dyads. The sources, function and shaping of seeding of neonate gut microbiota need, require more follow-up data and warrants further studies focused on microbial strain-level transference as well as on the potential microbiome functionality. Furthermore, more studies are needed to clarify how geographical location and differences in lifestyles could modify the effect of other perinatal factors on the initial microbial seeding. The consequences of differential gut microbiota acquisition and temporal patterns of microbiota evolution in our cohorts would require longitudinal studies assessing specific health outcomes.

## Data Availability Statement

The datasets presented in this study can be found in online repositories. The names of the repository/repositories and accession number(s) can be found below: https://www.ncbi.nlm.nih.gov/genbank/, PRJNA637167, https://www.ncbi.nlm.nih.gov/genbank/, PRJNA614975.

## Ethics Statement

The studies involving human participants were reviewed and approved by Ethics Committee of the Health Science Center, Xi’an Jiaotong University. Written informed consent to participate in this study was provided by the participants’ legal guardian/next of kin. The Spanish samples are from a MAMI birth cohort study which was conducted according to the guidelines of the Declaration of Helsinki, and it was approved by the Hospital Clínico Universitario de Valencia and the Bioethics Committee of CSIC (Consejo Superior de Investigaciones Científicas).

## Author Contributions

Conceptualization, BH and MCC. Methodology, YC and MS-R. Software, XC. Validation, QQ and JZ. Formal analysis, MC, MS-R, and YC. Investigation, QQ and XC. Resources, LZ. Data curation, YC and IG-M. Writing-original draft preparation, BH, MS-R and XC. Writing—review and editing, BH and MCC. Visualization, YC and MS-R. Supervision, BH and MCC. Project administration, LZ, BH, and MCC. Funding acquisition, BH and MCC. All authors agree to be accountable for the content of the work. All authors contributed to the article and approved the submitted version.

## Funding

This research was funded by National Natural Science Foundation of China (grant number 82173526, 81872633), and the APC was funded by 82173526. This work was also supported by the European Research Council under the European Union’s Horizon 2020 research and innovation program (ERC starting grant, no. 639226), as well to the ERC-NSFC cooperation program.

## Conflict of Interest

The authors declare that the research was conducted in the absence of any commercial or financial relationships that could be construed as a potential conflict of interest.

## Publisher’s Note

All claims expressed in this article are solely those of the authors and do not necessarily represent those of their affiliated organizations, or those of the publisher, the editors and the reviewers. Any product that may be evaluated in this article, or claim that may be made by its manufacturer, is not guaranteed or endorsed by the publisher.

## References

[B1] Adams R. I. Bateman A. C. Bik H. M. Meadow J. F. (2015). Microbiota of the Indoor Environment: A Meta-Analysis. Microbiome 3, 49. doi: 10.1186/s40168-015-0108-3 26459172 PMC4604073

[B2] Bäckhed F. Roswall J. Peng Y. Feng Q. Jia H. Kovatcheva-Datchary P.. (2015). Dynamics and Stabilization of the Human Gut Microbiome During the First Year of Life. Cell Host Microbe 17, 690–703. doi: 10.1016/j.chom.2015.05.012 25974306

[B3] Bailey L. C. Forrest C. B. Zhang P. Richards T. M. Livshits A. DeRusso P. A. (2014). Association of Antibiotics in Infancy With Early Childhood Obesity. JAMA Pediatr. 168, 1063–1069. doi: 10.1001/jamapediatrics.2014.1539 25265089

[B4] Benezra A. DeStefano J. Gordon J. I. (2012). Anthropology of Microbes. Proc. Natl. Acad. Sci. U. S. A 109, 6378–6381. doi: 10.1073/pnas.1200515109 22460792 PMC3340042

[B5] Benjamini Y. Yekutieli D. (2001). The Control of the False Discovery Rate in Multiple Testing Under Dependency. Ann. Statist. 29, 1165–1188. doi: 10.1214/aos/1013699998

[B6] Bokulich N. A. Kaehler B. D. Rideout J. R. Dillon M. Bolyen E. Knight R.. (2018). Optimizing Taxonomic Classification of Marker Gene Sequences. Microbiome 6 (1), 90. doi: 10.1186/s40168-018-0470-z 29773078 PMC5956843

[B7] Bolyen E. Rideout J. R. Dillon M. R. Bokulich N. A. Abnet C. C. Al-Ghalith G. A.. (2019). Reproducible, Interactive, Scalable and Extensible Microbiome Data Science Using QIIME 2. Nat. Biotechnol. 37, 1091. doi: 10.1038/s41587-019-0252-6 PMC7015180 31341288

[B8] Chen C. C. Chen K. J. Kong M. S. Chang H. J. Huang J. L. (2016). Alterations in the Gut Microbiotas of Children With Food Sensitization in Early Life. Pediatr. Allergy Immunol. 27, 254–262. doi: 10.1111/pai.12522 26663491

[B9] Chu D. M. Ma J. Prince A. L. Antony K. M. Seferovic M. D. Aagaard K. M. (2017). Maturation of the Infant Microbiome Community Structure and Function Across Multiple Body Sites and in Relation to Mode of Delivery. Nat. Med. 23, 314–326. doi: 10.1038/nm.4272 28112736 PMC5345907

[B10] Clemente J. C. Ursell L. K. Parfrey L. W. Knight R. (2012). The Impact of the Gut Microbiota on Human Health: An Integrative View. Cell 148, 1258–1270. doi: 10.1016/j.cell.2012.01.035 22424233 PMC5050011

[B11] Davis N. M. Proctor D. M. Holmes S. P. Relman D. A. Callahan B. J. (2018). Simple Statistical Identification and Removal of Contaminant Sequences in Marker-Gene and Metagenomics Data. Microbiome 6 (1), 226. doi: 10.1186/s40168-018-0605-2 30558668 PMC6298009

[B12] De Filippo C. Cavalieri D. Di Paola M. Ramazzotti M. Poullet J. B. Massart S.. (2010). Impact of Diet in Shaping Gut Microbiota Revealed by a Comparative Study in Children From Europe and Rural Africa. Proc. Natl. Acad. Sci. U. S. A. 107, 14691–14696. doi: 10.1073/pnas.1005963107 20679230 PMC2930426

[B13] Dominguez-Bello M. G. Costello E. K. Contreras M. Magris M. Hidalgo G. Fierer N.. (2010). Delivery Mode Shapes the Acquisition and Structure of the Initial Microbiota Across Multiple Body Habitats in Newborns. Proc. Natl. Acad. Sci. U. S. A. 107, 11971–11975. doi: 10.1073/pnas.1002601107 20566857 PMC2900693

[B14] Farhana L. Antaki F. Murshed F. Mahmud H. Judd S. L. Nangia-Makker P.. (2018). Gut Microbiome Profiling and Colorectal Cancer in African Americans and Caucasian Americans. World J. Gastrointest. Pathophysiol. 9 (2), 47–58. doi: 10.4291/wjgp.v9.i2.47 30283710 PMC6163128

[B15] Ferretti P. Pasolli E. Tett A. Asnicar F. Gorfer V. Fedi S.. (2018). Mother-To-Infant Microbial Transmission From Different Body Sites Shapes the Developing Infant Gut Microbiome. Cell Host Microbe 24, 133–145. doi: 10.1016/j.chom.2018.06.005 30001516 PMC6716579

[B16] García-Mantrana I. Alcántara C. Selma-Royo M. Boix-Amorós A. Dzidic M. Gimeno-Alcañiz J.. (2019). MAMI: A Birth Cohort Focused on Maternal-Infant Microbiota During Early Life. BMC Pediatr. 19, 140. doi: 10.1186/s12887-019-1502-y 31053102 PMC6498642

[B17] Grönlund M. M. Grześkowiak Ł. Isolauri E. Salminen S. (2011). Influence of Mother’s Intestinal Microbiota on Gut Colonization in the Infant. Gut Microbes 2, 227–233. doi: 10.4161/gmic.2.4.16799 21983067

[B18] Gueimonde M. Tölkkö S. Korpimäki T. Salminen S. (2004). New Real-Time Quantitative PCR Procedure for Quantification of Bifidobacteria in Human Fecal Samples. Appl. Environ. Microbiol. 70 (7), 4165–4169. doi: 10.1128/AEM.70.7.4165-4169.2004 15240297 PMC444799

[B19] Gupta V. K. Paul S. Dutta C. (2017). Geography, Ethnicity or Subsistence-Specific Variations in Human Microbiome Composition and Diversity. Front. Microbiol. 8, 1162. doi: 10.3389/fmicb.2017.01162 28690602 PMC5481955

[B20] Hansen R. Scott K. P. Khan S. Martin J. C. Berry S. H. Stevenson M.. (2015). First-Pass Meconium Samples From Healthy Term Vaginally-Delivered Neonates: An Analysis of the Microbiota. PloS One 10, e0133320. doi: 10.1371/journal.pone.0133320 26218283 PMC4517813

[B21] Jombart T. Ahmed I. (2011). Adegenet 1.3-1: New Tools for the Analysis of Genome-Wide SNP Data. Bioinformatics 27 (21), 3070–3071. doi: 10.1093/bioinformatics/btr521 21926124 PMC3198581

[B22] Kaehler B. D. Bokulich N. A. McDonald D. Knight R. Caporaso J. G. Huttley G.A. (2019). Species Abundance Information Improves Sequence Taxonomy Classification Accuracy. Nat. Commun. 10, 4643. doi: 10.1038/s41467-019-12669-6 31604942 PMC6789115

[B23] Klindworth A. Pruesse E. Schweer T. Peplies J. Quast C. Horn M.. (2013). Evaluation of General 16S Ribosomal RNA Gene PCR Primers for Classical and Next-Generation Sequencing-Based Diversity Studies. Nucleic Acids Res. 41, e1. doi: 10.1093/nar/gks808 22933715 PMC3592464

[B24] Koleva P. T. Kim J. S. Scott J. A. Kozyrskyj A. L. (2015). Microbial Programming of Health and Disease Starts During Fetal Life. Birth Defects Res. C. Embryo. Today 105, 265–277. doi: 10.1002/bdrc.21117 26663884

[B25] Kumar H. du Toit E. Kulkarni A. Aakko J. Linderborg K. M. Zhang Y.. (2016). Distinct Patterns in Human Milk Microbiota and Fatty Acid Profiles Across Specific Geographic Locations. Front. Microbiol. 7, 1619. doi: 10.3389/fmicb.2016.01619 27790209 PMC5061857

[B26] Lahti L. Shetty S. Microbiome R Package. Available at: http://microbiome.github.io.

[B27] Lax S. Sangwan N. Smith D. Larsen P. Handley K. M. Richardson M.. (2017). Bacterial Colonization and Succession in a Newly Opened Hospital. Sci. Transl. Med. 9, eaah6500. doi: 10.1126/scitranslmed.aah6500 28539477 PMC5706123

[B28] Lin A. Bik E. M. Costello E. K. Dethlefsen L. Haque R. Relman D. A.. (2013). Distinct Distal Gut Microbiome Diversity and Composition in Healthy Children From Bangladesh and the United States. PloS One 8, e53838. doi: 10.1371/journal.pone.0053838 23349750 PMC3551965

[B29] Liu C. J. Liang X. Niu Z. Y. Jin Q. Zeng X. Q. Wang W. X.. (2019). Is the Delivery Mode a Critical Factor for the Microbial Communities in the Meconium? EBioMedicine 49, 354–363. doi: 10.1016/j.ebiom.2019.10.045 31685443 PMC6945248

[B30] Magoč T. Salzberg S. L. (2011). FLASH: Fast Length Adjustment of Short Reads to Improve Genome Assemblies. Bioinformatics 27, 2957–2963. doi: 10.1093/bioinformatics/btr507 21903629 PMC3198573

[B31] Mallick H. Rahnavard A. McIver L. J. Ma S. Zhang Y. Nguyen L. H.. (2021). Multivariable Association Discovery in Population-Scale Meta-Omics Studies. PloS Comput. Biol. 17 (11), e1009442. doi: 10.1371/journal.pcbi.1009442 34784344 PMC8714082

[B32] Matsuki T. Yahagi K. Mori H. Matsumoto H. Hara T. Tajima S.. (2016). A Key Genetic Factor for Fucosyllactose Utilization Affects Infant Gut Microbiota Development. Nat. Commun. 7, 11939. doi: 10.1038/ncomms11939 27340092 PMC4931012

[B33] McMurdie P. J. Holmes S. (2013). Phyloseq: An R Package for Reproducible Interactive Analysis and Graphics of Microbiome Census Data. PloS One 8 (4), e61217. doi: 10.1371/journal.pone.0061217 23630581 PMC3632530

[B34] Michelsen C. F. Pedas P. Glaring M. A. Schjoerring J. K. Stougaard P. (2014). Bacterial Diversity in Greenlandic Soils as Affected by Potato Cropping and Inorganic Versus Organic Fertilization. Polar. Biol. 37, 61–71. doi: 10.1007/s00300-013-1410-9

[B35] Mikami K. Kimura M. Takahashi H. (2012). Influence of Maternal Bifidobacteria on the Development of Gut Bifidobacteria in Infants. Pharm. (Basel) 5, 629–642. doi: 10.3390/ph5060629 PMC3763658 24281665

[B36] Milani C. Duranti S. Bottacini F. Casey E. Turroni F. Mahony J.. (2017). The First Microbial Colonizers of the Human Gut: Composition, Activities, and Health Implications of the Infant Gut Microbiota. Microbiol. Mol. Biol. Rev. 81, e00036–e00017. doi: 10.1128/MMBR.00036-17 PMC5706746 29118049

[B37] Mueller N. T. Shin H. Pizoni A. Werlang I. C. Matte U. Goldani M. Z.. (2017). Delivery Mode and the Transition of Pioneering Gut-Microbiota Structure, Composition and Predicted Metabolic Function. Genes (Basel) 8, 364. doi: 10.3390/genes8120364 PMC5748682 29207565

[B38] Oksanen J. Blanchet F. G. Friendly M. Kindt R. Legendre P. McGlinn D.. (2019). “Vegan: Community Ecology Package,” in R Package Version 2, 5–5.

[B39] R Core Team. (2019). “R: A Language and Environment for Statistical Computing,” in R Foundation for Statistical Computing (Vienna, Austria).

[B40] Selma-Royo M. Calatayud A. M. García-Mantrana I. Parra-Llorca A. Escuriet R. Martínez-Costa C.. (2020). Perinatal Environment Shapes Microbiota Colonization and Infant Growth: Impact on Host Response and Intestinal Function. Microbiome 8, 167. doi: 10.1186/s40168-020-00940-8 33228771 PMC7685601

[B41] Sevelsted A. Stokholm J. Bønnelykke K. Bisgaard H. (2015). Cesarean Section and Chronic Immune Disorders. Pediatrics 135, e92–e98. doi: 10.1542/peds.2014-0596 25452656

[B42] Singh A. Mittal M. (2020). Neonatal Microbiome - A Brief Review. J. Matern. Fetal. Neonatal. Med. 33, 3841–3848. doi: 10.1080/14767058.2019.1583738 30835585

[B43] Sprockett D. Fukami T. Relman D. A. (2018). Role of Priority Effects in the Early-Life Assembly of the Gut Microbiota. Nat. Rev. Gastroenterol. Hepatol. 15, 197–205. doi: 10.1038/nrgastro.2017.173 29362469 PMC6813786

[B44] Stearns J. C. Zulyniak M. A. de Souza R. J. Campbell N. C. Fontes M. Shaikh M.. (2017). Ethnic and Diet-Related Differences in the Healthy Infant Microbiome. Genome Med. 9, 32. doi: 10.1186/s13073-017-0421-5 28356137 PMC5372248

[B45] Toda K. Hisata K. Satoh T. Katsumata N. Odamaki T. Mitsuyama E.. (2019). Neonatal Oral Fluid as a Transmission Route for Bifidobacteria to the Infant Gut Immediately After Birth. Sci. Rep. 9, 8692. doi: 10.1038/s41598-019-45198-9 31213639 PMC6582144

[B46] Turroni F. Duranti S. Milani C. Lugli G. A. van Sinderen D. Ventura M. (2019). *Bifidobacterium Bifidum*: A Key Member of the Early Human Gut Microbiota. Microorganisms 7, 544. doi: 10.3390/microorganisms7110544 PMC6920858 31717486

[B47] Wei T. Simko W. (2021). R Package ‘Corrplot’: Visualization of a Correlation Matrix (Version 0.92). Available at: https://github.com/taiyun/corrplot.

[B48] Wickham H. (2016). “Ggplot2: Elegant Graphics for Data Analysis,” in Mastering the Grammar (Springer-Verlag New York), 27–40.

[B49] Yang B. Yan S. Chen Y. Ross R. P. Stanton C. Zhao J.. (2020). Diversity of Gut Microbiota and Bifidobacterial Community of Chinese Subjects of Different Ages and From Different Regions. Microorganisms 8, 1108. doi: 10.3390/microorganisms8081108 PMC7464982 32722057

[B50] Yarza P. Richter M. Peplies J. Euzeby J. Amann R. Schleifer K. H.. (2008). The All-Species Living Tree Project: A 16S rRNA-Based Phylogenetic Tree of All Sequenced Type Strains. Syst. Appl. Microbiol. 31, 241–250. doi: 10.1016/j.syapm.2008.07.001 18692976

