# Supplementary material for: Influence of Geographical Location on Maternal-Infant Microbiota: Study in Two Populations From Asia and Europe
Source: Front Cell Infect Microbiol. 2022 Feb 4;11:663513. doi: 10.3389/fcimb.2021.663513 (PMC8855098; doi:10.3389/fcimb.2021.663513)
Supplement: Supplementary file 1 [file DataSheet_1.pdf]

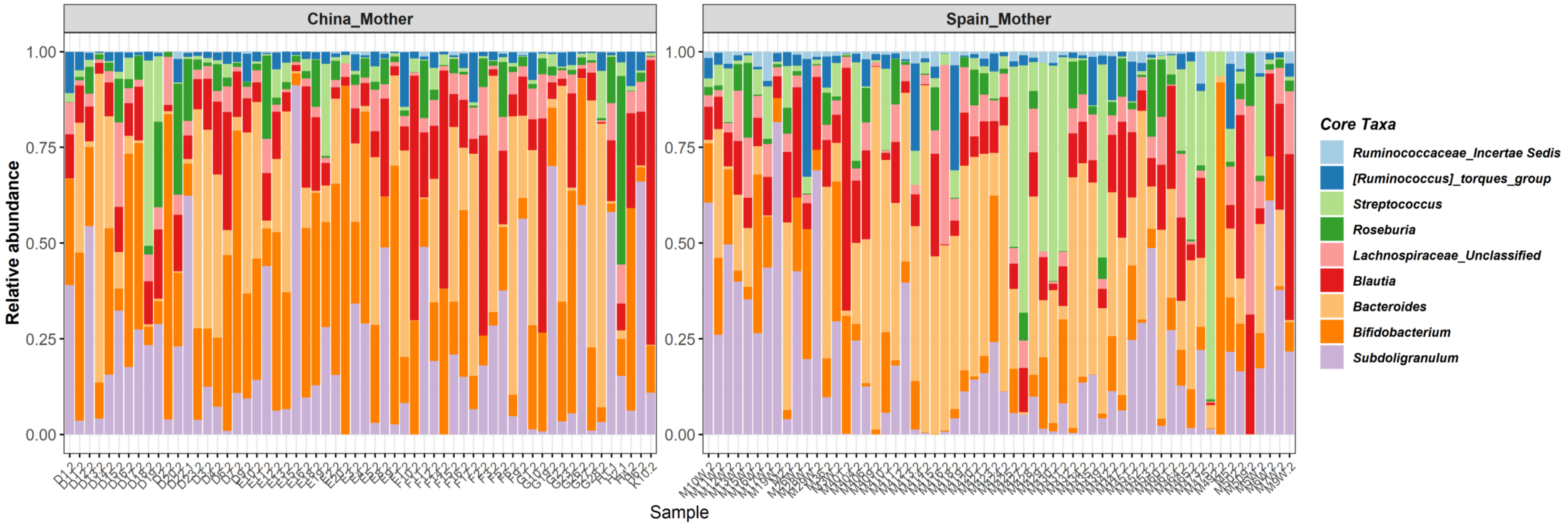

Supplementary Figure S1. Distribution of maternal gut microbiota core genera relative abundance at genus level according to location.

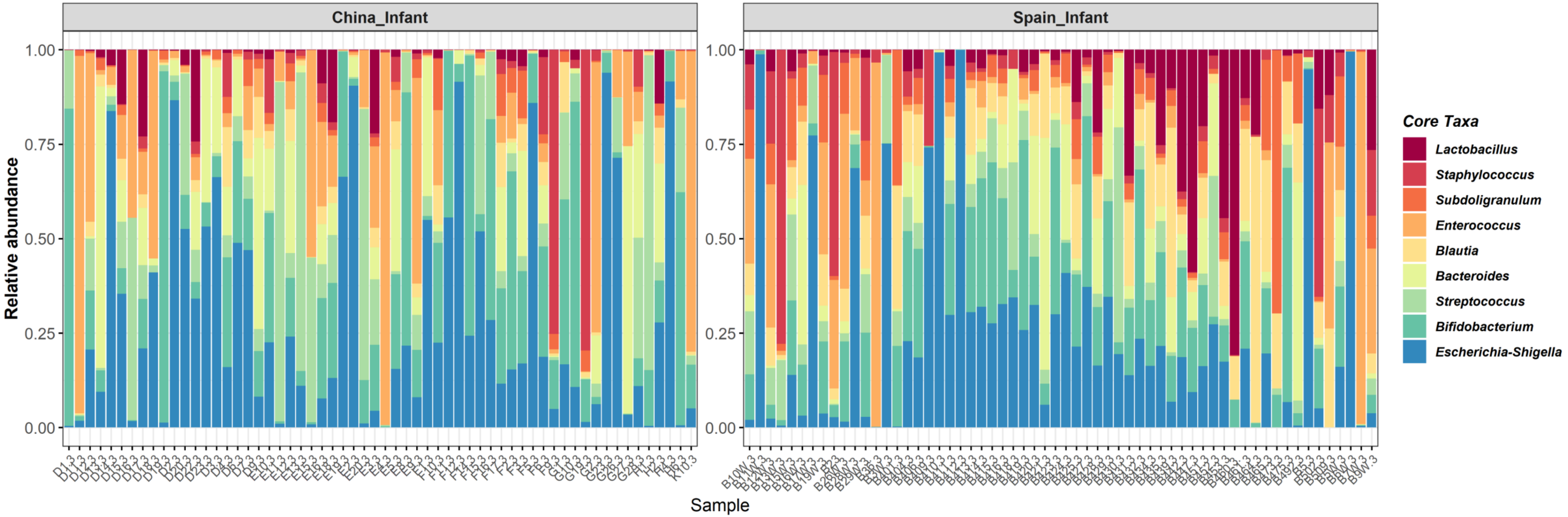

Supplementary Figure S2. Distribution of neonatal gut microbiota core genera relative abundance at genus level according to location.

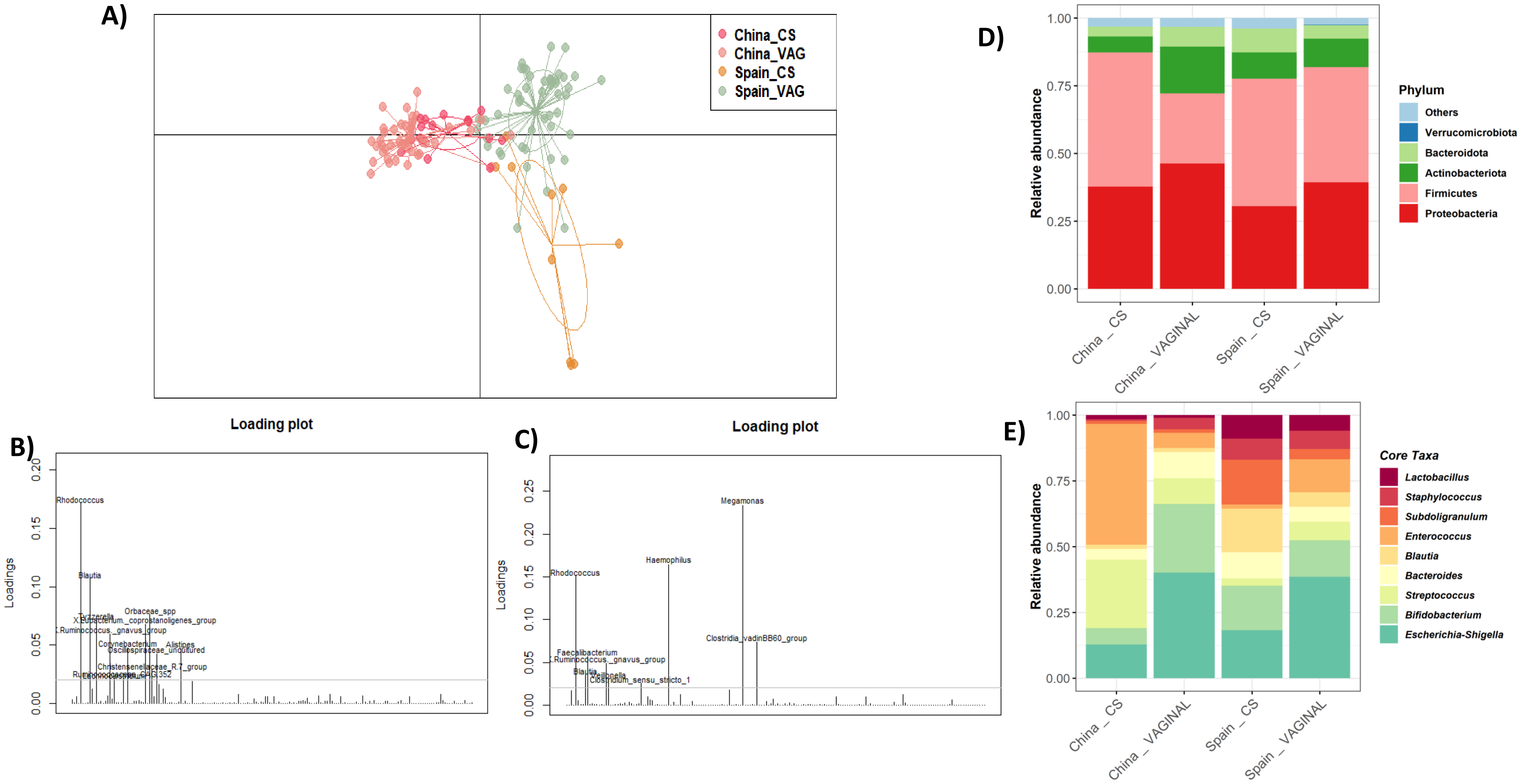

Supplementary Figure S3. Effect of the combination of delivery mode and geographical location in the neonatal gut microbiota. A) Discriminant analysis of principal components (DAPC) according both variables at genus level**. B-C)** Loading plots of the contribution of the variables in the both dimensions of DAPC, Dim. 1 (B) and Dim. 2 (C). **D-E)** Distribution of neoantal microbiota composition at phylum (B) and at genus level (C; only neonatal core genera are plotted) according to the resulted groups (delivery mode and location).
