# Supplementary material for: Influence of Geographical Location on Maternal-Infant Microbiota: Study in Two Populations From Asia and Europe
Source: Front Cell Infect Microbiol. 2022 Feb 4;11:663513. doi: 10.3389/fcimb.2021.663513 (PMC8855098; doi:10.3389/fcimb.2021.663513)
Supplement: Supplementary file 2 [file DataSheet_2.pdf]

Table S1. Relative abundance of core genera in both maternal and infant population according to nationality.

Taxa	General	China	Spain	Q-value
**Maternal population**				
Subdoligranulum	4.19 [0.9-10.28]	5.11 [1.92-15.56]	3.2 [0.37-9.55]	0.022
Bifidobacterium	3.99 [0.77-10.78]	7.36 [3.69-21.28]	0.83 [0.24-4.06]	<0.001
Bacteroides	3.26 [0.33-14.93]	1.77 [0.23-14.89]	3.76 [0.49-14.97]	0.527
Blautia	3.23 [1.27-5.92]	3.85 [1.84-7.5]	2.88 [0.74-4.58]	0.012
Lachnospiraceae_unclassified	1.39 [0.68-2.67]	1.37 [0.75-2.96]	1.47 [0.65-2.34]	0.313
Roseburia	0.62 [0.19-1.97]	0.79 [0.37-2.26]	0.48 [0.08-1.62]	0.012
Streptococcus	0.52 [0.19-1.46]	0.42 [0.18-1.12]	0.73 [0.19-2.33]	0.187
Ruminococcus_torques_group	0.47 [0.2-1.37]	0.77 [0.3-1.63]	0.35 [0.1-1.14]	0.012
Ruminococcaceae_incertae sedis	0.12 [0.05-0.33]	0.07 [0.04-0.14]	0.27 [0.08-0.59]	<0.001
**Infant population**				
Escherichia-Shigella	4.83 [1.44-19.87]	4.24 [1.12-39.61]	5.44 [1.63-17.71]	0.914
Bifidobacterium	4.72 [0.96-9.27]	4.82 [0.99-8.69]	4.61 [0.82-9.91]	0.926
Streptococcus	1.5 [0.34-3.81]	1.28 [0.35-4.41]	1.94 [0.29-3.69]	0.926
Bacteroides	1.24 [0.11-3.64]	0.99 [0.04-3.54]	1.32 [0.31-3.71]	0.275
Blautia	0.85 [0.15-3.33]	0.35 [0.11-1.16]	3.08 [0.69-4.61]	<0.001
Enterococcus	0.67 [0.12-3.41]	0.83 [0.15-4.54]	0.5 [0.09-1.93]	0.544
Subdoligranulum	0.56 [0.03-1.97]	0.24 [0.02-1.14]	1.27 [0.16-3.2]	0.001
Staphylococcus	0.35 [0.04-1.09]	0.17 [0.02-0.55]	0.61 [0.15-1.61]	0.001
Lactobacillus	0.18 [0.03-1.4]	0.08 [0.02-0.7]	0.78 [0.06-2.51]	0.001

Results are presented as median of relative abundance and interquartile range. Significant differences between geographical location in the core taxa were assessed by Mann-Whitney test on centered log ratio data.

Table S2. Results from Maaslin2 multivariate analysis in maternal microbiota

Genera	Coef ± SD	Prevalence	p-value	q-value
**Geographical location**				
Methylomonadaceae_Unclassified	6.20±0.58	48.3	p<0.001	q<0.001
Fenollaria	6.04±0.81	36.7	p<0.001	q<0.001
Psychrobacter	5.94±0.52	51.7	p<0.001	q<0.001
Faecalitalea	5.74±0.58	60	p<0.001	q<0.001
Finegoldia	5.65±0.79	29.2	p<0.001	q<0.001
Ezakiella	5.56±0.78	33.3	p<0.001	q<0.001
Prevotella	5.34±0.92	72.5	p<0.001	q<0.001
Campylobacter	5.21±0.82	30.8	p<0.001	q<0.001
Romboutsia	5.01±0.52	43.3	p<0.001	q<0.001
Eggerthella	4.77±0.72	76.7	p<0.001	q<0.001
Murdochiella	4.51±0.73	27.5	p<0.001	q<0.001
Oscillospiraceae_uncultured	4.37±0.6	95	p<0.001	q<0.001
Scardovia	4.32±0.45	48.3	p<0.001	q<0.001
Anaerovoracaceae_S5.A14a	4.28±0.61	28.3	p<0.001	q<0.001
Mobiluncus	3.87±0.63	28.3	p<0.001	q<0.001
Tyzzerella	3.65±0.6	90	p<0.001	q<0.001
Intestinibacter	3.58±0.49	36.7	p<0.001	q<0.001
Pseudarthrobacter	3.33±0.51	46.7	p<0.001	q<0.001
Negativicoccus	3.22±0.57	22.5	p<0.001	q<0.001
Orbaceae_Unclassified	3.13±0.69	17.5	p<0.001	q<0.001
Stenotrophobacter	3.1±0.37	41.7	p<0.001	q<0.001
Negativibacillus	2.99±0.51	31.7	p<0.001	q<0.001
Peptococcus	2.96±0.48	33.3	p<0.001	q<0.001
Alloprevotella	2.94±0.55	34.2	p<0.001	q<0.001
Fastidiosipila	2.62±0.56	18.3	p<0.001	q<0.001
Clostridia_UCG.014	2.59±0.84	81.7	0.003	0.020
Eubacterium_eligens_group	2.43±0.64	65	p<0.001	0.003
Ruminococcaceae_uncultured	2.41±0.68	73.3	0.001	0.006
Odoribacter	2.22±0.57	54.2	p<0.001	0.002
Peptostreptococcus	2.21±0.48	18.3	p<0.001	q<0.001
Megasphaera	2.12±0.62	70	0.001	0.008
Fusobacterium	2.05±0.66	26.7	0.002	0.018
Eubacterium_siraeum_group	1.74±0.62	47.5	0.006	0.039
Micromonosporaceae_Unclassified	1.72±0.53	25	0.001	0.012
Anaerotruncus	1.68±0.37	22.5	p<0.001	q<0.001
Atopobium	1.68±0.62	36.7	0.008	0.049
Lawsonella	1.65±0.59	35	0.006	0.040
Zixibacteria	1.64±0.52	21.7	0.002	0.018
Howardella	1.6±0.53	22.5	0.003	0.021
Ruminococcaceae_Unclassified	1.53±0.39	48.3	p<0.001	0.002
Peptococcaceae_uncultured	1.43±0.46	25	0.003	0.019
Adlercreutzia	1.42±0.39	20	p<0.001	0.004
Rhodospirillales_uncultured	1.42±0.36	22.5	p<0.001	0.002

Ruminococcaceae_Incertae_Sedis	1.37±0.44	96.7	0.003	0.020
Varibaculum	1.36±0.39	16.7	0.001	0.007
Holdemania	1.31±0.43	47.5	0.003	0.020
Uncultured	1.29±0.43	24.2	0.003	0.021
PeM15	1.2±0.44	18.3	0.008	0.048
Nocardioides	-0.5±0.18	11.7	0.007	0.045
Novosphingobium	-0.66±0.19	12.5	0.001	0.008
Rhodococcus	-0.89±0.29	19.2	0.003	0.021
Serratia	-1.06±0.33	20	0.002	0.013
Bacillus	-1.19±0.33	17.5	p<0.001	0.004
Lachnospiraceae_UCG.010	-1.25±0.31	25	p<0.001	0.001
Enhydrobacter	-1.3±0.31	22.5	p<0.001	0.001
Paraprevotella	-1.32±0.43	15.8	0.003	0.021
Colidextribacter	-1.43±0.32	25	p<0.001	q<0.001
Brevundimonas	-1.43±0.36	19.2	p<0.001	0.001
Weissella	-1.44±0.34	23.3	p<0.001	0.001
Lachnospiraceae_Unclassified	-1.44±0.44	99.2	0.001	0.011
Eubacterium_ruminantium_group	-1.48±0.33	26.7	p<0.001	q<0.001
Blautia	-1.49±0.45	99.2	0.001	0.011
Stenotrophomonas	-1.49±0.31	20	p<0.001	q<0.001
Acinetobacter	-1.6±0.4	27.5	p<0.001	0.001
Ruminococcaceae_DTU089	-1.64±0.34	26.7	p<0.001	q<0.001
Fusicatenibacter	-1.69±0.59	92.5	0.005	0.035
Lachnospiraceae_UCG.001	-1.8±0.52	31.7	0.001	0.006
Pseudomonas	-1.82±0.47	27.5	p<0.001	0.002
Hungatella	-1.87±0.35	30.8	p<0.001	q<0.001
Rothia	-1.92±0.58	66.7	0.001	0.011
Eubacterium_ventriosum_group	-1.96±0.62	81.7	0.002	0.015
Ruminococcus_torques_group	-2.01±0.49	96.7	p<0.001	0.001
Roseburia	-2.05±0.64	95.8	0.002	0.013
Corynebacterium	-2.08±0.48	19.2	p<0.001	q<0.001
Veillonella	-2.14±0.46	26.7	p<0.001	q<0.001
Turicibacter	-2.62±0.39	35	p<0.001	q<0.001
Enterobacteriaceae_Unclassified	-2.99±0.65	37.5	p<0.001	q<0.001
Sphingomonas	-3.09±0.59	42.5	p<0.001	q<0.001
Lachnoclostridium	-3.17±0.59	93.3	p<0.001	q<0.001
Bifidobacterium	-3.25±0.55	99.2	p<0.001	q<0.001
Collinsella	-3.27±0.69	90.8	p<0.001	q<0.001
Escherichia.Shigella	-3.44±0.73	96.7	p<0.001	q<0.001
Bilophila	-3.49±0.44	40	p<0.001	q<0.001
Megamonas	-3.72±0.77	35.8	p<0.001	q<0.001
Enterococcus	-3.73±0.67	65	p<0.001	q<0.001
Eubacterium_hallii_group	-3.94±0.44	39.2	p<0.001	q<0.001
Butyricicoccus	-4.43±0.3	49.2	p<0.001	q<0.001
Agathobacter	-4.44±0.85	93.3	p<0.001	q<0.001
Ruminococcaceae_CAG.352	-4.66±0.4	45.8	p<0.001	q<0.001

Alistipes	-5.16±0.63	41.7	p<0.001	q<0.001
Dialister	-5.68±0.54	46.7	p<0.001	q<0.001
Oscillospiraceae_UCG.002	-5.77±0.49	46.7	p<0.001	q<0.001
Dorea	-5.81±0.32	49.2	p<0.001	q<0.001
Ruminococcus_gnavus_group	-6.36±0.38	49.2	p<0.001	q<0.001
Eubacterium_coprostanoligenes_grou	-7.17±0.51	50.8	p<0.001	q<0.001
Faecalibacterium	-8.76±0.65	72.5	p<0.001	q<0.001
**Delivery mode**				
Lawsonella	-1.99±0.57	35	0.001	0.007
Orbaceae_Unclassified	-2.29±0.67	17.5	0.001	0.008
Negativicoccus	-1.85±0.55	22.5	0.001	0.010
Finegoldia	-2.47±0.77	29.2	0.002	0.013
Anaerovoracaceae_S5.A14a	-1.68±0.6	28.3	0.006	0.039
Mobiluncus	-1.63±0.61	28.3	0.009	0.054
Lactobacillales_P5D1.392	1.29±0.49	35	0.01	0.057
Campylobacter	-2.05±0.79	30.8	0.011	0.065
Muribaculaceae	-1.8±0.7	40	0.011	0.065
Prevotella	-2.28±0.9	72.5	0.012	0.068
Yersiniaceae_Unclassified	-0.68±0.27	13.3	0.014	0.075
Peptococcus	-1.16±0.47	33.3	0.015	0.080
Anaerostipes	1.23±0.5	93.3	0.015	0.081
Fenollaria	-1.91±0.79	36.7	0.017	0.084
Ezakiella	-1.82±0.76	33.3	0.018	0.086
Scardovia	1.05±0.44	48.3	0.018	0.087
Butyricicoccus	0.69±0.29	49.2	0.019	0.089
Monoglobus	1.49±0.68	90	0.032	0.133
Lachnospiraceae_Unclassified	0.88±0.42	99.2	0.039	0.159
**Maternal BMI**				
Eubacterium_eligens_group	-0.84±0.27	65	0.003	0.020
Lachnospira	-0.78±0.26	79.2	0.003	0.021
Oscillospiraceae_UCG.005	-0.93±0.31	83.3	0.003	0.023
Tyzzerella	-0.71±0.26	90	0.007	0.042
Clostridia_UCG.014	-0.96±0.36	81.7	0.009	0.053
Actinomyces	0.77±0.29	55.8	0.01	0.058
Odoribacter	-0.63±0.24	54.2	0.011	0.063
Eubacterium_xylanophilum_group	-0.6±0.23	48.3	0.012	0.066
Clostridia_vadinBB60_group	-0.55±0.22	30.8	0.015	0.078
Ruminococcus	-0.65±0.27	90.8	0.017	0.085
Ruminococcaceae_Unclassified	-0.4±0.17	48.3	0.02	0.093
Bacteroides	-0.8±0.35	98.3	0.022	0.102
Howardella	0.51±0.23	22.5	0.025	0.114
Psychrobacter	-0.5±0.22	51.7	0.027	0.120
Christensenellaceae_R.7_group	-0.7±0.32	90	0.029	0.126

Multivariate analysis was performed by Maaslin2 analysis including as covariables nationality, maternal BMI, delivery mode and maternal age. In the table, Spain nationality and vaginal delivery were considered as reference category for the coefficient's calculations. In the case of geographical location, those genera with q<0.05 are shown in the table. For delivery mode and maternal body mass index (BMI), those genera with differences q<0.15 are presented in the table.

Table S3. Results from Maaslin2 multivariate analysis in infant microbiota

Genera	Coef ± SD	Prevalence	p-value	q-value
**Geographical location**				
Ruminococcus_gnavus_group	-6.09±0.43	47.5	p<0.001	q<0.001
Dorea	-3.85±0.43	35.8	p<0.001	q<0.001
Psychrobacter	5.35±0.63	42.5	p<0.001	q<0.001
Ruminococcaceae_CAG.352	-4.93±0.61	38.3	p<0.001	q<0.001
Eubacterium_coprostanoligenes_group	-4.99±0.62	45	p<0.001	q<0.001
Eggerthella	5.05±0.68	59.2	p<0.001	q<0.001
Orbaceae_Unclassified	4.4±0.61	40	p<0.001	q<0.001
Alistipes	-4.25±0.59	35	p<0.001	q<0.001
Faecalitalea	4.31±0.6	40	p<0.001	q<0.001
Oscillospiraceae_UCG.002	-3.08±0.46	30	p<0.001	q<0.001
Scardovia	2.79±0.42	27.5	p<0.001	q<0.001
Corynebacterium	-3.86±0.59	34.2	p<0.001	q<0.001
Methylomonadaceae_Unclassified	4.03±0.62	37.5	p<0.001	q<0.001
Rhodococcus	-3.65±0.6	44.2	p<0.001	q<0.001
Tyzzerella	4.76±0.79	65.8	p<0.001	q<0.001
Butyricicoccus	-2.54±0.42	30.8	p<0.001	q<0.001
Turicibacter	-3.34±0.56	33.3	p<0.001	q<0.001
Romboutsia	3.01±0.52	29.2	p<0.001	q<0.001
Yersiniaceae_Unclassified	3.41±0.59	41.7	p<0.001	q<0.001
Dialister	-3.74±0.66	27.5	p<0.001	q<0.001
Lachnospiraceae_ND3007_group	3.77±0.69	46.7	p<0.001	q<0.001
Enterobacter	-4.37±0.8	85	p<0.001	q<0.001
Finegoldia	3.86±0.71	25.8	p<0.001	q<0.001
Eubacterium._hallii_group	-2.54±0.48	30.8	p<0.001	q<0.001
Stenotrophobacter	1.71±0.33	26.7	p<0.001	q<0.001
Fenollaria	3.3±0.63	35.8	p<0.001	q<0.001
Clostridioides	2.81±0.54	25	p<0.001	q<0.001
Megamonas	-2.41±0.5	29.2	p<0.001	q<0.001
Oscillospiraceae_uncultured	3.73±0.79	70	p<0.001	q<0.001
Anaerotruncus	1.58±0.35	15	p<0.001	q<0.001
Bilophila	-1.82±0.42	20.8	p<0.001	0.001
Negativibacillus	1.72±0.4	20	p<0.001	0.001
Sphingomonadaceae_Unclassified	-2.22±0.52	19.2	p<0.001	0.001
Intestinibacter	1.89±0.45	23.3	p<0.001	0.001
Eubacterium._eligens_group	2.79±0.7	36.7	p<0.001	0.002
Colidextribacter	-1.56±0.39	16.7	p<0.001	0.003
Clostridia_vadinBB60_group	-2.3±0.6	25.8	p<0.001	0.003
Gardnerella	2.33±0.61	20.8	p<0.001	0.003
Anaerovoracaceae_S5.A14a	1.75±0.46	14.2	p<0.001	0.004
Lachnospira	2.12±0.57	40	p<0.001	0.005
Lachnospiraceae_CAG.56	2.24±0.62	33.3	p<0.001	0.007
Ezakiella	2.28±0.63	20.8	p<0.001	0.007
Ruminococcaceae_Incertae_Sedis	2.55±0.71	50.8	p<0.001	0.007

Prevotella	3.29±0.92	63.3	p<0.001	0.007
Holdemania	0.92±0.26	11.7	0.001	0.007
Eubacterium._xylanophilum_group	2.3±0.65	35.8	0.001	0.007
Murdochiella	1.42±0.4	15	0.001	0.008
Rothia	-2.31±0.66	73.3	0.001	0.009
Kocuria	1.93±0.56	40.8	0.001	0.010
Nocardioides	-1.79±0.52	24.2	0.001	0.011
Veillonella	-2.21±0.65	37.5	0.001	0.011
Butyricicoccaceae_UCG.009	0.83±0.25	12.5	0.001	0.013
Lachnospiraceae_UCG.010	-0.76±0.23	12.5	0.001	0.017
Coprobacillus	1.02±0.32	14.2	0.002	0.019
Eubacterium._siraeum_group	1.64±0.52	24.2	0.002	0.024
Novosphingobium	-1.79±0.57	27.5	0.002	0.025
Zixibacteria	1.21±0.39	18.3	0.002	0.026
Eubacterium._ruminantium_group	-0.87±0.29	10.8	0.003	0.034
Alloprevotella	1.81±0.61	39.2	0.004	0.036
**Delivery mode**				
Escherichia.Shigella	3.04±0.69	97.5	p<0.001	q<0.001
Veillonella	-2.73±0.63	37.5	p<0.001	0.001
Eubacterium_brachy_group	-0.81±0.31	13.3	0.010	0.092
Atopobium	-1.32±0.55	21.7	0.018	0.126
Lachnospiraceae_GCA.900066575	-1.04±0.43	23.3	0.018	0.127
**Maternal BMI**				
Lachnospiraceae_NK4A136_group	-1.34±0.35	76.7	p<0.001	0.003
Erysipelotrichaceae_UCG.003	-1.04±0.3	56.7	0.001	0.011
Ruminococcus	-1.09±0.34	76.7	0.002	0.023
Fusicatenibacter	-0.91±0.3	75.8	0.003	0.034
Lactobacillus	-1.03±0.34	94.2	0.003	0.035
Phascolarctobacterium	-0.84±0.29	49.2	0.004	0.043
Monoglobus	-0.9±0.31	67.5	0.004	0.043
Lachnospiraceae_ND3007_group	-0.84±0.29	46.7	0.005	0.048
Ruminococcus._torques_group	-0.81±0.32	80.8	0.011	0.096
Anaerostipes	-0.8±0.31	70.8	0.012	0.102
Desulfovibrio	-0.59±0.23	33.3	0.013	0.103
Tyzzerella	-0.85±0.34	65.8	0.013	0.103
Lachnospiraceae_CAG.56	-0.67±0.27	33.3	0.013	0.104
Faecalitalea	-0.64±0.26	40	0.014	0.107
Oscillospiraceae_NK4A214_group	-0.82±0.34	60	0.016	0.119
Blautia	-0.69±0.28	94.2	0.016	0.121
Pseudarthrobacter	-0.68±0.28	42.5	0.018	0.126

Multivariate analysis was performed by Maaslin2 analysis including as covariables nationality, maternal BMI and delivery mode. In the table, Spain nationality and vaginal delivery were considered as reference category for the coefficient's calculations. In the case of geographical location, those genera with q<0.05 are shown in the table. For delivery mode and maternal body mass index (BMI), those genera with differences q<0.15 are presented in the table.
